# Repurposing antibiotic resistance surveillance data to support treatment of recurrent infections in a remote setting

**DOI:** 10.1038/s41598-023-50008-4

**Published:** 2024-01-29

**Authors:** Will Cuningham, Shalinie Perera, Sonali Coulter, Zhiqiang Wang, Steven Y. C. Tong, Teresa M. Wozniak

**Affiliations:** 1 Menzies School of Health Research, Charles Darwin University, Darwin, NT Australia; 2 Centre for Neonatal and Paediatric Infection, St. George’s University of London, London, SW17 0RE UK; 3 Western Diagnostic Pathology, Perth, WA Australia; 4 Medication Services Queensland, Prevention Division, Department of Health, Brisbane, QLD Australia; 5 Victorian Infectious Diseases Service, The Royal Melbourne Hospital at the Peter Doherty Institute for Infection and Immunity, Melbourne, VIC Australia; 6 Department of Infectious Diseases, The University of Melbourne at the Peter Doherty Institute for Infection and Immunity, Melbourne, VIC Australia; 7 Australian e-Health Research Centre CSIRO, Brisbane, QLD Australia

**Keywords:** Antibiotics, Epidemiology

## Abstract

In northern Australia, a region with limited access to healthcare and a substantial population living remotely, antibiotic resistance adds to the complexity of treating infections. Focussing on *Escherichia coli* urinary tract infections (UTIs) and *Staphylococcus aureus* skin & soft tissue infections (SSTIs) captured by a northern Australian antibiotic resistance surveillance system, we used logistic regression to investigate predictors of a subsequent resistant isolate during the same infection episode. We also investigated predictors of recurrent infection. Our analysis included 98,651 *E. coli* isolates and 121,755 *S. aureus* isolates from 70,851 patients between January 2007 and June 2020. Following an initially susceptible *E. coli* UTI, subsequent recovery of a cefazolin (8%) or ampicillin (13%) -resistant isolate during the same infection episode was more common than a ceftriaxone-resistant isolate (2%). For an initially susceptible *S. aureus* SSTI, subsequent recovery of a methicillin-resistant isolate (8%) was more common than a trimethoprim-sulfamethoxazole-resistant isolate (2%). For UTIs and SSTIs, prior infection with a resistant pathogen was a strong predictor of both recurrent infection and resistance in future infection episodes. This multi-centre study demonstrates an association between antibiotic resistance and an increased likelihood of recurrent infection. Particularly in remote areas, a patient’s past antibiograms should guide current treatment choices since recurrent infection will most likely be at least as resistant as previous infection episodes. Using population-level surveillance data in this way can also help clinicians decide if they should switch antibiotics for patients with ongoing symptoms, while waiting for diagnostic results.

## Introduction

Often the main purpose of infection surveillance systems is to track population-level trends, aggregating individual data by region. Given the large amount of antibiotic susceptibility data captured by microbiological diagnostic labs, there is an opportunity to consider clinically relevant questions at the patient level over time.

In this study, we used a longitudinal microbiological dataset to ask: (1) if additional isolates are recovered during an infection episode, what are the predictors that those isolates are antibiotic-resistant; (2) what are the predictors that additional infection episodes (i.e., recurrent infection) occur; (3) if recurrent infection occurs, what are the predictors that the recurrent infection is resistant. We hypothesised that resistance would be more likely for first-line oral agents, due to frequency of use^1,2^, and that resistant organisms would be more likely to be associated with recurrent infection, due to failure of initial empirical therapy^3–5^. Specifically, we explored these hypotheses in *E. coli* isolated from urinary tract specimens and *S. aureus* isolated from skin & soft tissue specimens given the high rates of resistance in these organisms^6,7^.

Urinary tract infections (UTI) and skin & soft tissue infections (SSTI) occur regularly in both the community and hospital settings, and are associated with frequent antibiotic use^8–14^. In remote northern Australia, the selective pressure created by frequent infections and antibiotic use is further complicated by limited diagnostic capacity and under-resourced antimicrobial stewardship activities^15^. Compared with antibiotic-susceptible infections, resistant infections often lead to worse health outcomes, increasing hospital length of stay and associated healthcare costs^16^. Additionally, a significant consequence of antibiotic resistance is recurrence of infection, potentially because of a delay in administering the appropriate antibiotic therapy^17–19^.

SSTIs are particularly prevalent in this setting^20–24^. *Staphylococcus aureus* demonstrates an increasing prevalence of resistance to β-lactam antibiotics over time and at levels much higher in remote communities (up to 50% methicillin-resistant [MRSA]) than elsewhere in Australia (approx. 15% MRSA)^25–27^. Furthermore, uropathogens such as *Escherichia coli* are almost 20% cefazolin-resistant and 35% trimethoprim-resistant in northern Australia, with resistance rates increasing 1–3% annually^28^.

For all patients with suspected UTIs, local treatment guidelines recommend microbiological testing before giving antibiotics (especially for more severe infections such as cystitis/pyelonephritis)^29,30^. For certain SSTIs (e.g., impetigo), some guidelines recommend testing only if the patient is not responding to empirical therapy^29^, while others suggest to check for MRSA immediately^30^. The high prevalence of SSTIs in this setting means that it is not always practical to send a swab to the lab. Furthermore, in the absence of point-of-care diagnostics and limited access to timely microbiology laboratory services, antibiotic therapy is usually empirical in remote northern Australia^29–32^.

Knowledge of any previous antibiograms helps to inform subsequent treatment decisions. To this end, data such as those analysed in this study can be used to support real-time clinical decisions in under-resourced settings, in addition to acting as a traditional surveillance system that helps to optimise treatment guidelines and stewardship interventions^33–35^.

## Methods

### Study setting

We used data from a laboratory-based, phenotypic antibiotic resistance surveillance system covering most of the population in northern Australia (January 2007–June 2020), which has been described in detail elsewhere^27,28^. This study included data from major tertiary centres, regional and rural hospitals as well as community clinics across northern Australia (i.e., the entire Northern Territory and the area above the Tropic of Capricorn in Western Australia and Queensland).

### Microbiological data

We analysed all *E. coli* isolates from urinary tract specimens and all *S. aureus* isolates from skin & soft tissue specimens, and their corresponding antibiotic susceptibilities. Only one isolate per specimen was included in the analysis. A unique number was used to track each patient and their isolates over time. No additional clinical data or laboratory results (such as urine white cell count) were available, preventing us distinguishing infection from colonisation.

Depending on the pathology provider, susceptibility testing was done using VITEK 2 (bioMérieux) and/or disc-diffusion techniques with results interpreted using either CLSI (2021) or EUCAST (2020) standards. Participating laboratories were accredited under regularly audited national testing guidelines (National Association of Testing Authorities), ensuring a high concordance and reproducibility of susceptibility results between different laboratories.

Based on clinical importance, treatment guidelines and available data^29–31^, our analysis of resistance in *E. coli* isolates included resistance to ampicillin, amoxicillin-clavulanate, cefazolin, ceftriaxone, ciprofloxacin, nitrofurantoin, trimethoprim or trimethoprim-sulfamethoxazole. In *S. aureus* isolates, analyses included resistance to penicillin, methicillin (i.e., resistance to oxacillin or flucloxacillin), erythromycin, clindamycin or trimethoprim-sulfamethoxazole.

### Definitions

The following section defines principles used to prepare the data, describes the criteria for inclusion in each analysis (represented visually in Figure S1) and explains model predictor variables.

Analysis 1: predictors of resistance to each antibiotic during an infection episode.

Analysis 2/2b: predictors of recurrent infection.

Analysis 3/3b: predictors of a resistant recurrent infection.

[Note – analyses 2 and 3 used a predictor variable combining resistance to four first-line antibiotics (i.e., resistant to zero [fully susceptible], one, two, three or four of amoxicillin-clavulanate, cefazolin, nitrofurantoin & trimethoprim for *E. coli*, and clindamycin, methicillin, penicillin & trimethoprim-sulfamethoxazole for *S. aureus*). We also ran separate models for each antibiotic in Analyses 2b and 3b.]

Infection episode: at least one *E. coli* isolate from a urinary tract specimen (i.e., a UTI episode), or at least one *S. aureus* isolate from a skin or soft tissue specimen (i.e., a SSTI episode). Isolates within 30 days of a previous isolate of the same organism were defined as belonging to the same infection episode^36–38^.

Index isolate: the first isolate of each infection episode.

Duration of infection episode: number of days from the index isolate to last isolate of the infection episode (for Analysis 1, duration was censored at the first resistant isolate if one occurred).

Resistance during an infection episode (Analysis 1): following a susceptible index isolate, a resistant isolate (to the same antibiotic) during that infection episode. Only infection episodes with at least two isolates could be included.

History of resistance (Analysis 1): at least one isolate in a previous infection episode resistant to the same antibiotic.

Resistant infection episode (Analysis 2/2b/3/3b): an infection episode with a resistant isolate at any point during the infection episode (otherwise susceptible if it only contained susceptible isolates).

Recurrent infection (Analysis 2/2b): at least one infection episode in the six months after an infection episode^39^.

History of recurrent infection: at least one infection episode in the six months before an infection episode.

Resistant recurrent infection (Analysis 3/3b): a resistant index isolate in at least one of the infection episodes meeting the recurrent infection definition.

### Statistical analyses

We used logistic regression in all analyses. Unlike Analysis 1, Analyses 2/2b and 3/3b included infection episodes with only one isolate, however, patients with only one isolate recorded in the entire dataset were excluded as they could not contribute to any of the analyses.

All analyses were stratified by infection type (i.e., *E. coli* UTI and *S. aureus* SSTI), and results were presented as odds ratios (OR). Additionally, Analysis 1 was adjusted for sex, age group, the duration of the infection episode, number of isolates in the infection episode, history of recurrent infection, history of resistance and presence of co-resistance to at least one of the other antibiotics. Analyses 2/2b and 3/3b were adjusted for sex, age group, number of infection episodes and history of recurrent infection.

We used Stata 16.1 and R (via RStudio 1.3) to clean and analyse the data^40,41^. All the methods were carried out in accordance with the National Statement on Ethical Conduct in Human Research (2007). The experimental protocol (including a waiver of informed consent for use of retrospective data) was approved by the Human Research Ethics Committee of the Northern Territory Department of Health and Menzies School of Health Research (HREC-2018-3084) and the Queensland Health Public Health Act 2005 (Section 280).

## Results

### Patient characteristics and trends

**Figure 1:**
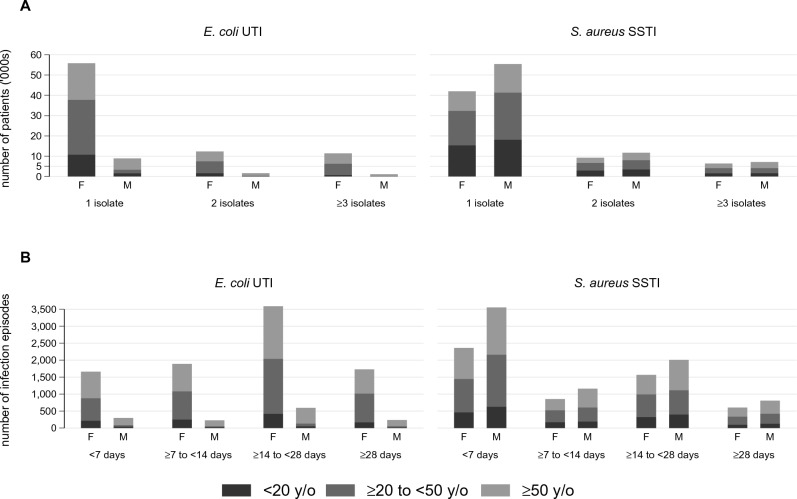
(**A**) Number of patients in the original dataset with 1 isolate, 2 isolates and ≥ 3 isolates (note: patients with only one isolate excluded from all analyses), by sex and age group. (**B**) Distribution of infection episode durations (excluding infection episodes with only one isolate), by sex and age group. Note: F: female; M: male (a large number [2,837] of *S. aureus* SSTIs from the Territory Pathology dataset had a duration < 7 days [Territory Pathology didn’t provide age/sex data and so doesn’t appear in the figure]).

There were 243,239 patients with 167,969 *E. coli* (urinary tract) and 230,014 *S. aureus* (skin & soft tissue) isolates. We excluded 172,388 patients (71%) with only one isolate in the dataset (69,318 *E. coli* and 108,259 *S. aureus* [5,189 patients with one of each]) from all analyses, leaving 98,651 *E. coli* and 121,755 *S. aureus* isolates from 70,851 patients (Tables 1, S1). Most patients did not have a subsequent isolate (Fig. 1), but we were unable to quantify the number of patients whose infection resolved (e.g., due to antibiotic therapy) or the number who had an ongoing infection.

**Table 1:** Descriptive characteristics of data.

	*E. coli* UTI	*S. aureus* SSTI
Number of patients	30,932	43,909
Number of males [%]:females [%]*	2,913 [10]:25,689 [90]	20,700 [55]:16,983 [45]
Age (years), median [IQR]*	43 [25–66]	34 [15–53]
Number of infection episodes [%]	84,585	88,999
Number of infection episodes per patient, median [IQR]	2 [2, 3]	2 [1–3]
Duration (days) of infection episodes, median [IQR]^§^	16 [7–25]	6 [2–18]
Duration (days) between infection episodes, median [IQR]	273 [99–716]	319 [109–828]
Number of isolates [%]	98,651	121,755
Number of isolates per patient, median [IQR]	2 [2, 3]	2 [2, 3]
Number of isolates per infection episode, median [IQR]	1 [1–1]	1 [1–1]
Duration (days) between isolates, median [IQR]	162 [41–552]	145 [25–584]
Antibiotics included in analysis [percentage of isolates tested]^‡^	amp [97]; amc [99]; cfz [99]; ctx [98]; cip [50]; nit [7]; trm [7]; sxt [7]	cln [85]; ery [99]; mth [87]; pen [84]; sxt [96]

*At first isolate collected per unique patient; sex unknown for 2,330 (8%) and 6,226 (14%) patients with a UTI and SSTI respectively (age/sex not available from Territory Pathology).

^§^Excluding infection episodes with only one isolate.

^‡^amp: ampicillin; amc: amoxicillin-clavulanate; cfz: cefazolin; ctx: ceftriaxone; cip: ciprofloxacin; cln: clindamycin; ery: erythromycin; mth: methicillin (flucloxacillin in Western Diagnostic Pathology data (up until end of 2014 only), flucloxacillin in Pathology Queensland data, oxacillin in Territory Pathology data); nit: nitrofurantoin; pen: penicillin; trm: trimethoprim; sxt: trimethoprim-sulfamethoxazole.

Note: all statistics calculated after excluding patients with only one isolate; 3,990 patients had both ≥ 1 UTI and ≥ 1 SSTI.

Patients with UTIs and patients with SSTIs both had a median of two infection episodes (interquartile ranges: 2–3 and 1–3 respectively), however the median duration of a UTI was longer (16 [7–25] days) than the duration of an SSTI (6 [2–18] days) (Table 1, Fig. 1). Patients with a UTI were mostly female (10% male, 90% female) and a median of 43 [25–66] years old. Patients with an SSTI were more evenly distributed in sex (55% male, 45% female) and a median of 34 [15–53] years old (Tables 1, S2).

**Figure 2:**
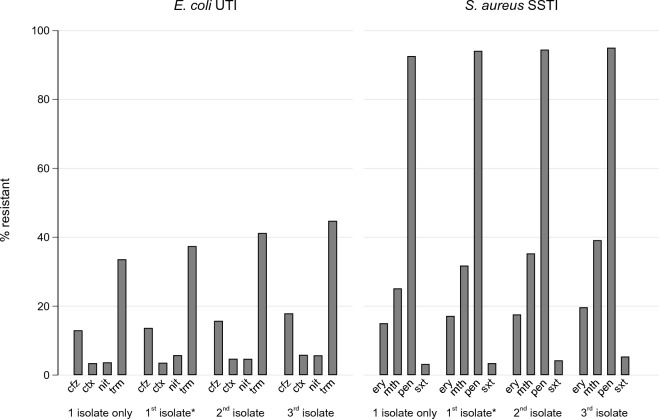
Percentage of isolates antibiotic resistant by isolate number per patient (up to the 3rd isolate), as well as patients with only one isolate for comparative purposes (note: these were excluded from all analyses). *Excluding patients with only 1 isolate. Note: cfz: cefazolin; ctx: ceftriaxone; ery: erythromycin; mth: methicillin (i.e., oxacillin or flucloxacillin); nit: nitrofurantoin; pen: penicillin; trm: trimethoprim; sxt: trimethoprim-sulfamethoxazole.

The percentage of isolates resistant increased with each isolate recovered per patient (Fig. 2). Table 2 shows susceptibilities by sequential isolates per patient. The percentage resistant was higher if the previous isolate was resistant. For example, 14% (4,226/30,483) of patients’ first *E. coli* isolates were resistant to cefazolin, and for these patients, 50% (1,940/3,872) were resistant to cefazolin in the second isolate. Conversely, for the 86% (26,257/30,483) of first *E. coli* isolates that were cefazolin-susceptible, only 10% (2,519/24,390) were resistant to cefazolin in the second isolate. Resistance to one antibiotic also increased the likelihood of co-resistance to another antibiotic (Figure S2).

**Table 2:** Number (%) of isolates resistant/susceptible tiered through first three isolates (by patient*), by antibiotic.

		First isolate		Second isolate		Third isolate		All^‡^
*E. coli* UTI	cefazolin	R	4226 (14)	R	1940 (50)	R	625 (65)	16,741 (17)
						S	331 (35)	
				S	1932 (50)	R	182 (19)	
						S	791 (81)	
		S	26,257 (86)	R	2519 (10)	R	528 (43)	81,109 (83)
						S	696 (57)	
				S	21,871 (90)	R	1041 (10)	
						S	9038 (90)	
		All^		R	4491 (16)	R	2404 (18)	as above
				S	24,031 (84)	S	11,022 (82)	as above
	ceftriaxone	R	1111 (4)	R	680 (68)	R	273 (81)	5051 (5)
						S	66 (19)	
				S	324 (32)	R	21 (13)	
						S	140 (87)	
		S	29,036 (96)	R	644 (2)	R	157 (53)	91,496 (95)
						S	137 (47)	
				S	26,153 (98)	R	318 (3)	
						S	11,810 (97)	
		All^		R	1335 (5)	R	776 (6)	as above
				S	26,865 (95)	S	12,446 (94)	as above
	nitrofurantoin	R	136 (6)	R	48 (36)	R	8 (38)	385 (5)
						S	13 (62)	
				S	85 (64)	R	1 (3)	
						S	29 (97)	
		S	2183 (94)	R	61 (3)	R	15 (42)	6709 (95)
						S	21 (58)	
				S	2120 (97)	R	31 (4)	
						S	853 (96)	
		All^		R	109 (5)	R	56 (6)	as above
				S	2208 (95)	S	919 (94)	as above
	trimethoprim	R	867 (37)	R	669 (77)	R	256 (81)	3074 (43)
						S	61 (19)	
				S	198 (23)	R	28 (33)	
						S	56 (67)	
		S	1452 (63)	R	287 (20)	R	81 (65)	4022 (57)
						S	44 (35)	
				S	1162 (80)	R	72 (16)	
						S	376 (84)	
		All^		R	956 (41)	R	437 (45)	as above
				S	1363 (59)	S	539 (55)	as above
*S. aureus* SSTI	erythromycin	R	7319 (17)	R	3791 (54)	R	1051 (66)	22,986 (19)
						S	544 (34)	
				S	3185 (46)	R	286 (21)	
						S	1064 (79)	
		S	36,530 (83)	R	3342 (10)	R	707 (48)	98,599 (81)
						S	765 (52)	
				S	30,224 (90)	R	1109 (10)	
						S	10,507 (90)	
		All^		R	7140 (18)	R	3162 (20)	As above
				S	33,451 (82)	S	12,909 (80)	As above
	methicillin	R	12,691 (31)	R	8159 (74)	R	2583 (79)	38,088 (36)
						S	688 (21)	
				S	2832 (26)	R	351 (31)	
						S	768 (69)	
		S	27,635 (69)	R	3935 (17)	R	1149 (66)	68,199 (64)
						S	602 (34)	
				S	19,325 (83)	R	1176 (16)	
						S	6115 (84)	
		All^		R	12,111 (35)	R	5272 (39)	As above
				S	22,195 (65)	S	8198 (61)	As above
	penicillin	R	35,367 (94)	R	31,270 (97)	R	11,934 (97)	97,048 (94)
						S	319 (3)	
				S	1058 (3)	R	281 (72)	
						S	112 (28)	
		S	2290 (6)	R	1183 (58)	R	447 (92)	5734 (6)
						S	41 (8)	
				S	841 (42)	R	124 (39)	
						S	193 (61)	
		All^		R	32,499 (94)	R	12,816 (95)	as above
				S	1903 (6)	S	671 (5)	as above
	SXT	R	1408 (3)	R	747 (57)	R	222 (71)	5401 (5)
						S	90 (29)	
				S	556 (43)	R	27 (12)	
						S	196 (88)	
		S	40,166 (97)	R	890 (2)	R	157 (42)	111,208 (95)
						S	220 (58)	
				S	35,633 (98)	R	400 (3)	
						S	13,182 (97)	
		All^		R	1672 (4)	R	836 (5)	as above
				S	37,456 (96)	S	14,673 (95)	as above

*Isolates resistant/susceptible in all first/second/third isolates as recorded over each patient’s entire observation time (i.e., per patient). Second and third isolates presented as subset of previously recorded susceptibilities (denominator of percentages equal to number of isolates with a susceptibility recorded). Excludes patients with only one isolate.

^‡^Isolates resistant/susceptible including all isolates at once (i.e., not per order in patient observation time).

^Isolates resistant/susceptible in all second and third isolates as opposed to conditional on previous susceptibilities.

Note: R: resistant; S: susceptible; SXT: trimethoprim-sulfamethoxazole.

### Predictors of resistance during an infection episode (Analysis 1)

Our first analysis focussed on predictors of resistance in subsequent isolates within the same infection episode for patients with non-resolved index-susceptible infection episodes (Table 3).

**Table 3:** Predictors of subsequent resistant isolates during an index-susceptible infection episode (Analysis 1).

			OR (95% CI); p-value*
*E. coli* UTI^	Resistance	Ceftriaxone	1 [reference]
		Ciprofloxacin	1.24 (0.81–1.86); 0.30
		Amoxicillin-clavulanate	5.67 (4.38–7.43); < 0.01
		Cefazolin	5.68 (4.40–7.44); < 0.01
		Ampicillin	97.3 (67.07–143.63); < 0.01
	Sex	Female	1 [reference]
		Male	0.78 (0.59–1.02); 0.08
	Age group	≥ 20 to < 50 years old	1 [reference]
		< 20 years old	1.34 (1.04–1.71); 0.02
		≥ 50 years old	1.46 (1.28–1.68); < 0.01
	Duration of infection episode (days)^A^		1.00 (0.99–1.01); 0.39
	Number of isolates^B^		0.77 (0.65–0.91); < 0.01
	History of recurrent infection^C^		0.91 (0.80–1.04); 0.16
	History of resistance^D^		2.62 (2.28–3.00); < 0.01
	Resistance to ≥ 1 other antibiotic^E^		35.25 (26.09–48.56); < 0.01
*S. aureus* SSTI	Resistance	Trimethoprim-sulfamethoxazole	1 [reference]
		Clindamycin	2.70 (2.00–3.69); < 0.01
		Erythromycin	3.04 (2.27–4.12); < 0.01
		Methicillin	7.74 (5.70–10.63); < 0.01
	Sex	Female	1 [reference]
		Male	0.79 (0.67–0.95); 0.01
	Age group	≥ 20 to < 50 years old	1 [reference]
		< 20 years old	0.80 (0.59–1.06); 0.13
		≥ 50 years old	1.03 (0.86–1.24); 0.75
	Duration of infection episode (days)^A^		1.04 (1.03–1.05); < 0.01
	Number of isolates^B^		0.46 (0.38–0.57); < 0.01
	History of recurrent infection^C^		1.54 (1.29–1.84); < 0.01
	History of resistance^D^		4.41 (3.69–5.29); < 0.01
	Resistance to ≥ 1 other antibiotic^E^		6.50 (5.29–8.03); < 0.01

^A^Days from index isolate to last isolate or first resistant isolate (whichever came first).

^B^Censored at last isolate or first resistant isolate (whichever came first).

^C^ ≥ 1 infection episode in the previous 6 months.

^D^ ≥ 1 isolate in a previous infection episode resistant to the same antibiotic.

^E^Resistance to ≥ 1 of the other antibiotics included in the model at any point during the infection episode^B^.

*Model degrees of freedom (residual): *E. coli* = 16,271; *S. aureus* = 13,138.

^When excluding sex & age from the model, ORs for *Resistance* variable: Nitrofurantoin = 1, Ceftriaxone = 1.06 (0.56–2.29), Ciprofloxacin = 1.46 (0.75–3.21), Cefazolin = 5.18 (2.81–10.96), Amoxicillin-clavulanate = 5.37 (2.91–11.35), Trimethoprim-sulfamethoxazole = 6.41 (2.99–14.94), Trimethoprim = 7.21 (3.36–16.87), Ampicillin = 72.81 (37.63–159.22) – all other values remained similar.

Note: nitrofurantoin, trimethoprim and trimethoprim-sulfamethoxazole not included in *E. coli* model as sex/age not available; penicillin not included in *S. aureus* model due to insufficient data (i.e., very few infection episodes began as penicillin-susceptible).

#### *E. coli* UTIs

Patients who had at least one resistant isolate in a previous UTI had a greater than 2.5-fold higher likelihood (OR: 2.62 [95% confidence interval: 2.28–3.00]) of resistance to the same antibiotic during the current UTI (Table 3). Those aged 20–50 years old had a lower likelihood of resistance during a UTI (< 20 years old: 1.34 [1.04–1.71]; 20–50: 1.00 (reference); ≥ 50: 1.46 [1.28–1.68]).

Subsequent *E. coli* isolates were unlikely to be resistant to ceftriaxone (165/9,997 [2%]) and ciprofloxacin (111/5,158 [2%]) compared with other antibiotics (Tables 3, S3). Compared with ceftriaxone, subsequent *E. coli* isolates were more likely to be resistant to amoxicillin-clavulanate (5.67 [4.38–7.43]), cefazolin (5.68 [4.40–7.44]) and ampicillin (97.3 [67.07–143.63]). After excluding sex and age from the model (due to missing data), we could include nitrofurantoin (least likely to be resistant during an infection episode, 21/1,028 [2%]), trimethoprim-sulfamethoxazole (6.41 [2.99–14.94] relative to nitrofurantoin) and trimethoprim (7.21 [3.36–16.87] relative to nitrofurantoin); the ORs for other antibiotics did not change substantively.

#### *S. aureus* SSTIs

The odds of resistance during an SSTI were almost 4.5-fold higher (4.41 [3.69–5.29]) for those who had at least one resistant isolate (to the same antibiotic) in a previous SSTI (Table 3). Males had a lower likelihood of resistance during an SSTI (0.79 [0.67–0.95]).

Subsequent *S. aureus* isolates were unlikely to be resistant to trimethoprim-sulfamethoxazole (279/15,447 [2%]) (Tables 3, S3). Compared with trimethoprim-sulfamethoxazole, subsequent *S. aureus* isolates were more likely to be resistant to clindamycin (2.70 [2.00–3.69]), erythromycin (OR: 3.04 [2.27–4.12]) and methicillin (7.74 [5.70–10.63]).

### Predictors of recurrent infection (Analysis 2/2b)

Our second analysis focussed on predictors of recurrent infection (i.e., at least one infection episode within the subsequent 6 months) for patients with index-susceptible or index-resistant infection episodes (i.e., all infection episodes) (Tables 4, S4).

**Table 4:** Predictors of recurrent infection and of a resistant recurrent infection (Analysis 2 and 3 respectively).

			OR (95% CI); p-value*	
			Recurrent vs no recurrent infection (Analysis 2)	Resistant vs susceptible recurrent infection (Analysis 3)
*E. coli* UTI^	Resistance	Resistant to 0 antibiotics	1 [reference]	1 [reference]
		Resistant to 1 antibiotic	1.18 (1.08–1.29); < 0.01	9.86 (8.62–11.29); < 0.01
		Resistant to 2 antibiotics	1.08 (1.01–1.16); 0.02	11.90 (10.68–13.26); < 0.01
	Sex	Female	1 [reference]	1 [reference]
		Male	1.44 (1.30–1.59); < 0.01	1.46 (1.24–1.73); < 0.01
	Age group	≥ 20 to < 50 years old	1 [reference]	1 [reference]
		< 20 years old	0.95 (0.86–1.05); 0.32	0.95 (0.77–1.17); 0.66
		≥ 50 years old	1.15 (1.09–1.21); < 0.01	1.12 (1.02–1.24); 0.02
	Number of infection episodes^A^		1.09 (1.09–1.10); < 0.01	1.02 (1.01–1.03); < 0.01
	History of recurrent infection^B^		1.55 (1.48–1.63); < 0.01	0.90 (0.82–0.99); 0.03
*S. aureus* SSTI	Resistance	Resistant to 0 antibiotics	1 [reference]	1 [reference]
		Resistant to 1 antibiotic	1.16 (1.00–1.33); 0.04	27.71 (21.02–36.65); < 0.01
		Resistant to 2 antibiotics	1.38 (1.19–1.59); < 0.01	50.89 (35.85–73.44); < 0.01
		Resistant to 3 antibiotics	1.70 (1.43–2.02); < 0.01	71.19 (37.46–153.59); < 0.01
		Resistant to 4 antibiotics	2.19 (1.60–3.02); < 0.01	24.79 (10.83–71.71); 0.08
	Sex	Female	1 [reference]	1 [reference]
		Male	1.07 (1.01–1.13); 0.02	0.83 (0.65–1.05); 0.12
	Age group	≥ 20 to < 50 years old	1 [reference]	1 [reference]
		< 20 years old	0.73 (0.68–0.79); < 0.01	1.42 (0.94–2.20); 0.10
		≥ 50 years old	1.29 (1.21–1.38); < 0.01	0.71 (0.54–0.92); 0.01
	Number of infection episodes^A^		1.10 (1.09–1.12); < 0.01	1.01 (0.97–1.07); 0.57
	History of recurrent infection^B^		1.42 (1.34–1.50); < 0.01	0.89 (0.70–1.14); 0.36

^A^Number of infection episodes per patient (including the current infection episode).

^B^ ≥ 1 infection episode in the previous 6 months.

*Model degrees of freedom (residual): *E. coli* = 27,212; *S. aureus* = 21,315 for Analysis 2; *E. coli* = 12,560; *S. aureus* = 9,001 for Analysis 3.

^When excluding sex & age from the model, ORs for *Resistance* variable: 1 antibiotic = 1.20 (1.10–1.30), 11.02 (9.72–12.51); 2 antibiotics = 1.11 (1.04–1.18), 12.71 (11.44–14.14); 3 antibiotics = 1.57 (1.09–2.25), 53.64 (26.26–129.04); 4 antibiotics = 2.29 (1.08–5.17), 55.30 (16.02–347.57) for Analysis 2 and 3 respectively – all other values remained similar.

Note: antibiotics included were amoxicillin-clavulanate, cefazolin, nitrofurantoin & trimethoprim for *E. coli* and clindamycin, methicillin, penicillin & trimethoprim-sulfamethoxazole for *S. aureus.*

#### *E. coli* UTIs

A higher number of UTIs overall and at least one UTI in the previous six months increased the likelihood of recurrent UTI (1.09 [1.09–1.10] and 1.55 [1.48–1.63] respectively) (Table 4). The likelihood of recurrent UTI was higher for males (1.44 [1.30–1.59]) and older age groups (< 20 years old: 0.95 [0.86–1.05]; 20–50: 1.00 (reference); ≥ 50: 1.15 [1.09–1.21]).

The number of fully susceptible UTIs that had recurrent UTI was 15,913/41,117 (39%), while this was 2,105/4,745 (44%), 3,292/7,429 (44%), 117/255 (46%) and 25/47 (53%) for UTIs resistant to one to four antibiotics respectively (Table S3). The likelihood of recurrent UTI was higher for UTIs resistant to one and two antibiotics (OR: 1.18 [1.08–1.29] and 1.08 [1.01–1.16] respectively) compared with fully susceptible UTIs, and, after excluding sex and age from the model (due to missing data), for UTIs resistant to three and four antibiotics (1.57 [1.09–2.25] and 2.29 (1.08–5.17) respectively) (Table 4).

We also ran separate models for each antibiotic (Analysis 2b). For all antibiotics, compared with susceptible UTIs, a greater percentage of resistant UTIs had recurrent UTI (Table S3), and this translated to a significant OR for amoxicillin-clavulanate (1.08 [1.02–1.15]), ampicillin (1.10 [1.04–1.15]), cefazolin (1.10 [1.03–1.17]), ceftriaxone (1.48 [1.32–1.67]) and ciprofloxacin (1.53 [1.35–1.73]) (Table S4). The same associations as in Analysis 2 regarding sex, age, the number of UTIs and history of recurrent UTIs were evident.

#### *S. aureus* SSTIs

A higher number of SSTIs overall and at least one SSTI in the previous six months increased the likelihood of recurrent SSTI (1.10 [1.09–1.12] and 1.42 [1.34–1.50] respectively) (Table 4). The likelihood of recurrent SSTI was higher for males (1.07 [1.01–1.13]) and older age groups (< 20 years old: 0.73 [0.68–0.79]; 20–50: 1.00 (reference); ≥ 50: 1.29 [1.21–1.38]).

The number of fully susceptible SSTIs that had recurrent SSTI was 2,080/5,623 (37%), while this was 34%, 40%, 50% and 59% for SSTIs resistant to one to four antibiotics respectively (Table S3). The likelihood of recurrent SSTI was higher for SSTIs resistant to one, two, three and four antibiotics (OR: 1.16 [1.00–1.33], 1.38 [1.19–1.59], 1.70 [1.43–2.02] and 2.19 [1.01–3.02] respectively) compared with fully susceptible SSTIs (Table 4).

We also ran separate models for each antibiotic (Analysis 2b). For all antibiotics, compared with susceptible SSTIs, a greater percentage of resistant SSTIs had recurrent SSTI (Table S3), and this translated to a significant OR for most antibiotics (Table S4). The same associations as in Analysis 2 regarding sex, age, the number of SSTIs and history of recurrent SSTIs were evident.

### Predictors of a resistant recurrent infection (Analysis 3/3b)

Our third analysis focussed on predictors of a resistant recurrent infection (i.e., an index-resistant isolate in at least one of the infection episodes meeting the recurrent infection definition) for patients with infection episodes that had recurrent infection (Tables 4, S4).

#### *E. coli* UTIs

The number of fully susceptible UTIs that had a resistant recurrent UTI was 2,162/15,913 (14%), while this was 1,375/2,105 (65%), 2,242/3,292 (68%), 103/117 (88%) and 22/25 (88%) for UTIs resistant to one to four antibiotics respectively (Table S3). Correspondingly, the likelihood of a resistant recurrent UTI was higher for UTIs resistant to one and two antibiotics (OR: 9.86 [8.62–11.29] and 11.90 [10.68–13.26] respectively) compared with fully susceptible UTIs (Table 4). The likelihood of a resistant recurrent UTI was higher for males (1.46 [1.24–1.73]) and patients aged ≥ 50 years old compared with those 20–50 years old (1.12 [1.02–1.24]) (Tables 4, S4).

#### *S. aureus* SSTIs

The number of fully susceptible SSTIs that had a resistant recurrent SSTI was 848/2,080 (41%), while this was 10,243/10,719 (96%), 6,355/6,506 (98%), 1,201/1,242 (97%) and 202/209 (97%) for SSTIs resistant to one to four antibiotics respectively (Table S3). Correspondingly, the likelihood of a resistant recurrent SSTI was higher for SSTIs resistant to one, two, three and four antibiotics (OR: 27.71 [21.02–36.65], 50.89 [35.85–73.44], 71.19 [37.46–153.59] and 24.79 [10.83–71.71] respectively) compared with fully susceptible SSTIs (Table 4). The likelihood of a resistant recurrent SSTI was lower for patients aged ≥ 50 years old compared with those 20–50 years old (0.71 [0.54–0.92]).

## Discussion

### Overview of results

We are not aware of any other studies that have used this approach to investigate the relationship between antibiotic resistance and recurrent infection, repurposing population-level surveillance data. We found that previous resistant infection episodes were consistently the strongest predictor of both recurrent infection and resistance in future infection episodes, highlighting that antibiograms of previous infection episodes should guide current treatment choices. Furthermore, we quantified the likelihood of resistance to commonly used antibiotics during an infection episode, helping to inform remote clinicians’ treatment of patients with a continuing infection episode when culture-directed therapy is not readily accessible in a timely manner. The results of this study are largely supported by literature from other settings describing recurrent infections and associated factors, including an increased risk due to antibiotic resistance^42–51^.

### Predictors of resistance during an infection episode (Analysis 1)

Antibiotics are frequently used empirically in the community setting^1,7^. Compared with intravenous agents used for more severe infections in the hospital setting, we found that it was more likely that subsequent isolates of an index-susceptible UTI were resistant to first-line oral antibiotics. In our study, resistance to trimethoprim was quite common (43% excluding patients with only one isolate), which may influence resistance to trimethoprim-sulfamethoxazole. Furthermore, the high prevalence of SSTIs (particularly due to MRSA in this setting) and associated use of first-line trimethoprim-sulfamethoxazole could impact resistance profiles of Gram-negative bacteria^25,26^. However, in line with current recommendations, we found that trimethoprim-sulfamethoxazole remains a very good option for the treatment of SSTIs^52^, and there are other antibiotics such as nitrofurantoin that could be used to treat UTIs if resistance to trimethoprim (and other empirical agents like cephalexin) continues to increase^53,54^. UTI treatment guidelines generally remain appropriate given the current rates of resistance (amoxicillin-clavulanate and cephalexin < 20%), and continue to be revised for antibiotics with resistance of concern (i.e., trimethoprim)^28^. Promisingly, antibiotic use seems to be regularly in accordance with treatment guidelines in this region^55^.

While sex did not have a statistically significant effect for patients with UTIs (despite UTIs being more prevalent among females), males with SSTIs had a decreased likelihood of subsequent resistant isolates. Conversely, while age was not a modifying factor for patients with SSTIs, patients aged 20–50 years old had a decreased likelihood of resistance during a UTI. The association between age and UTIs has been observed previously^8,9,56^, but the relationship between resistance and age and sex in our models needs to be investigated further as it would be affected by comorbidities and other factors not captured in our dataset.

Finally, for both UTIs and SSTIs, perhaps the best predictor of resistance during an infection episode was a previous resistant infection episode. It is important to have access to previous antibiograms and culture-directed therapy as soon as possible after a subsequent infection, and the patient should be monitored more closely for non-resolving infection due to resistance.

### Predictors of recurrent infection (Analysis 2/2b)

For both UTIs and SSTIs, patients were more likely to have recurrent infection after a resistant infection episode. This association became stronger for infection episodes resistant to multiple antibiotics. The occurrence of recurrent infection episodes after resistant infection episodes may be because of a failure to clear the infection due to suboptimal antibiotic therapy, the ability of resistant organisms to persist in colonising microbial flora (e.g., in the case of UTIs), or the abundance of resistant organisms in the environment (e.g., in the case of SSTIs).

Common to both UTIs and SSTIs, recent prior infection episodes are strong predictors of recurrent infection, indicating that the approach to patient management (including antibiotic therapy based on susceptibility testing) needs to be revised to interrupt the cycle of recurrent infection^5^. Finally, for both UTIs and SSTIs, the likelihood of recurrent infection increased with age and for males.

### Predictors of a resistant recurrent infection (Analysis 3/3b)

If a patient had a recurrent infection after a resistant infection episode, it was very likely that that recurrent infection would also be resistant^3,17–19^. A resistant recurrent infection was less common after a susceptible infection episode, but culture-directed therapy may still be useful in this scenario.

### Limitations

We considered every isolate as representing infection. Since we had no additional clinical data (including choice of antibiotic therapy), laboratory results or information on patient history, we could not distinguish infection from colonisation. Furthermore, we based our definition of an infection episode on the timing of specimen collection. Although an assumption, the interval of 30 days since previous specimen collection was informed by the literature^36–38^.

There are some ways in which our dataset would not have captured every infection episode. Since observation time did not begin from birth, the first infection episode recorded for each patient in our dataset may not have been that patient’s first ever infection. Furthermore, we could not censor a patient’s observation time in the event they became available for inclusion in our dataset after January 2007 or became unavailable for inclusion before June 2020 (e.g., due to change in residence). We also didn’t have data on infection episodes of patients who didn’t get a specimen collected and/or organism cultured. Detection bias might have been further amplified by treatment failure increasing the likelihood of culturing and retrieving resistant isolates. Finally, the surveillance system does not include specimens from patients in private healthcare facilities. These missing data and inability to adjust for follow-up time may have impacted our results. More sophisticated study designs and statistical approaches (e.g., generalised linear models) may be useful in addressing such limitations, as well as adjusting for time-varying covariables and residual confounding due to lack of clinical exposure data (e.g., antibiotic therapy and comorbidities)^57,58^.

Comparison of data from participating laboratories should be approached with some caution due to the use of either CLSI or EUCAST methods. However, both methods are internationally recognised, and differences are well documented. For the bacteria and antibiotics in this study, any differences in breakpoints are minimal and we believe our results were not substantively impacted. Additionally, isolates were sourced from a combination of tertiary hospitals as well as community clinics, and while this may have some impact on our analysis, the overall implications of our findings were not substantively affected.

## Conclusions

Using a large amount of patient-level data over time, we have addressed questions that are relevant to daily clinical practice. Our results can assist clinicians in remote settings select the most appropriate antibiotic treatment until culture-directed therapy is possible. Aggregated antibiotic resistance surveillance systems are very useful in understanding the high-level epidemiological picture, but we have demonstrated these comprehensive resources can be used in other informative ways. Additional linkage with clinical patient data would enable more robust analyses.

### Supplementary Information

Supplementary Information.

## Data Availability

Due to data sharing agreements with the data custodians, the data used and analysed in this study are not publicly available. Requests for access to the data for research purposes can be sent to DS4AMR@csiro.au and will be considered on a case-by-case basis.

## Abbreviations

UTI: Urinary tract infection
SSTI: Skin & soft tissue infection
*S. aureus*: *Staphylococcus aureus*
MRSA: Methicillin-resistant *Staphylococcus aureus*
*E. coli*: *Escherichia coli*
OR: Odds ratio
IQR: Interquartile range
