# Supplementary material for: Repurposing antibiotic resistance surveillance data to support treatment of recurrent infections in a remote setting
Source: Sci Rep. 2024 Jan 29;14:2414. doi: 10.1038/s41598-023-50008-4 (PMC10825221; doi:10.1038/s41598-023-50008-4)
Supplement: Supplementary file 1 — Supplementary Information. [file 41598_2023_50008_MOESM1_ESM.docx]

Table S1. Descriptive characteristics of data, by pathology provider.

|  | Data source | *E. coli* UTI | *S. aureus* SSTI |
| --- | --- | --- | --- |
| Number of patients [%^˄^] | Western Diagnostic Pathology | 12,514 [40] | 11,362 [26] |
|  | Territory Pathology | 2,322 [8] | 6,190 [14] |
|  | Pathology Queensland | 16,096 [52] | 26,357 [60] |
|  | Total | 30,932 [100] | 43,909 [100] |
| Number of males [%]:females [%]* | Western Diagnostic Pathology | 710 [6]:11,803 [94] | 5,676 [50]:5,685 [50] |
|  | Territory Pathology | NA | NA |
|  | Pathology Queensland | 2,203 [14]:13,886 [86] | 15,024 [56]:11,298 [44] |
|  | Total | 2,913 [10]:25,689 [90] | 20,700 [55]:16,983 [45] |
| Age (years), median [IQR]* | Western Diagnostic Pathology | 35 [22-53] | 30 [10-48] |
|  | Territory Pathology | NA | NA |
|  | Pathology Queensland | 52 [28-74] | 36 [16-56] |
|  | Total | 43 [25-66] | 34 [15-53] |
| Number of infection episodes [%^˄^] | Western Diagnostic Pathology | 40,011 [47] | 24,527 [28] |
|  | Territory Pathology | 5,630 [7] | 11,864 [13] |
|  | Pathology Queensland | 38,944 [46] | 52,607 [59] |
|  | Total | 84,585 [100] | 88,999 [100] |
| Number of infection episodes per patient, median [IQR] | Western Diagnostic Pathology | 2 [2-4] | 2 [2-3] |
|  | Territory Pathology | 2 [2-3] | 2 [1-2] |
|  | Pathology Queensland | 2 [1-3] | 2 [1-2] |
|  | Total | 2 [2-3] | 2 [1-3] |
| Duration (days) of infection episodes, median [IQR]^§^ | Western Diagnostic Pathology | 18 [10-26] | 14 [6-23] |
|  | Territory Pathology | 12 [2-23] | 3 [1-13] |
|  | Pathology Queensland | 15 [7-24] | 6 [2-19] |
|  | Total | 16 [7-25] | 6 [2-18] |
| Duration (days) between infection episodes, median [IQR] | Western Diagnostic Pathology | 272 [102-700] | 382 [133-947] |
|  | Territory Pathology | 243 [87-648] | 263 [91-686] |
|  | Pathology Queensland | 281 [98-746] | 299 [103-800] |
|  | Total | 273 [99-716] | 319 [109-828] |
| Number of isolates [%^˄^] | Western Diagnostic Pathology | 45,234 [46] | 30,965 [25] |
|  | Territory Pathology | 7,102 [7] | 18,807 [16] |
|  | Pathology Queensland | 46,315 [47] | 71,983 [59] |
|  | Total | 98,651 [100] | 121,755 [100] |
| Number of isolates per patient, median [IQR] | Western Diagnostic Pathology | 3 [2-4] | 2 [2-3] |
|  | Territory Pathology | 2 [2-3] | 2 [2-3] |
|  | Pathology Queensland | 2 [2-3] | 2 [2-3] |
|  | Total | 2 [2-3] | 2 [2-3] |
| Number of isolates per infection episode, median [IQR] | Western Diagnostic Pathology | 1 [1-1] | 1 [1-1] |
|  | Territory Pathology | 1 [1-1] | 1 [1-2] |
|  | Pathology Queensland | 1 [1-1] | 1 [1-1] |
|  | Total | 1 [1-1] | 1 [1-1] |
| Duration (days) between isolates, median [IQR] | Western Diagnostic Pathology | 187 [55-582] | 292 [75-833] |
|  | Territory Pathology | 97 [22-421] | 47 [3-315] |
|  | Pathology Queensland | 144 [32-539] | 129 [23-541] |
|  | Total | 162 [41-552] | 145 [25-584] |
| Antibiotics included in analysis [percentage of isolates tested]^‡^ | Western Diagnostic Pathology | amp [99]; amc [99]; cfz [99]; ctx [99]; cip [11] | cln [45]; ery [99]; mth [51]; pen [99]; sxt [99] |
|  | Territory Pathology | amp [64]; amc [100]; cfz [99]; ctx [99]; cip [99]; nit [99]; trm [99]; sxt [99] | cln [99]; ery [99]; mth [99]; sxt [99] |
|  | Pathology Queensland | amp [99]; amc [99]; cfz [98]; ctx [96]; cip [80] | cln [99]; ery [99]; mth [99]; pen [99]; sxt [93] |
|  | Total | amp [97]; amc [99]; cfz [99]; ctx [98]; cip [50]; nit [7]; trm [7]; sxt [7] | cln [85]; ery [99]; mth [87]; pen [84]; sxt [96] |
| Date range | Western Diagnostic Pathology | Jan/2007 – Apr/2019 | |
|  | Territory Pathology | Jan/2012 – Jun/2020 | |
|  | Pathology Queensland | Jan/2008 – Dec/2019 | |
|  | Total | Jan/2007 – Jun/2020 | |

* at first isolate collected per unique patient; age/sex not available from TP (<1% missing from WDP & PQ)

^˄^ percentage of total

^§^ excluding infection episodes with only one isolate

^‡^ amp: ampicillin; amc: amoxicillin-clavulanate; cfz: cefazolin; ctx: ceftriaxone; cip: ciprofloxacin; cln: clindamycin; ery: erythromycin; mth: methicillin (flucloxacillin in Western Diagnostic Pathology data (up until end of 2014 only), flucloxacillin in Pathology Queensland data, oxacillin in Territory Pathology data); nit: nitrofurantoin; pen: penicillin; trm: trimethoprim; sxt: trimethoprim-sulfamethoxazole

Note: all statistics calculated after excluding patients with only one isolate; 1,373, 433 and 2,184 patients had both ≥1 UTI and ≥1 SSTI in WDP, TP and PQ data, respectively.

Table S2. Number of index isolates* (column %, row %), by sex, age group, antibiotic and susceptibility.

|  | | | | Susceptible | Resistant | Total |
| --- | --- | --- | --- | --- | --- | --- |
| *E. coli* UTI | Amoxicillin-clavulanate | sex | Female | 60263 (87%, 84%) | 11951 (80%, 17%) | 72214 (86%, 100%) |
|  |  |  | Male | 5053 (7%, 76%) | 1578 (11%, 24%) | 6631 (8%, 100%) |
|  |  |  | Total | 69534 (100%, 82%) | 14957 (100%, 18%) | 84491 (100%, 100%) |
|  |  | age group | <20 | 6945 (10%, 86%) | 1152 (8%, 14%) | 8097 (10%, 100%) |
|  |  |  | ≥20 to <50 | 29956 (43%, 84%) | 5735 (38%, 16%) | 35691 (42%, 100%) |
|  |  |  | ≥50 | 28426 (41%, 81%) | 6646 (44%, 19%) | 35072 (42%, 100%) |
|  |  |  | Total | 69534 (100%, 82%) | 14957 (100%, 18%) | 84491 (100%, 100%) |
|  | Ampicillin | sex | Female | 32670 (88%, 45%) | 39603 (87%, 55%) | 72273 (88%, 100%) |
|  |  |  | Male | 3141 (8%, 47%) | 3490 (8%, 53%) | 6631 (8%, 100%) |
|  |  |  | Total | 37282 (100%, 45%) | 45295 (100%, 55%) | 82577 (100%, 100%) |
|  |  | age group | <20 | 3547 (10%, 44%) | 4562 (10%, 56%) | 8109 (10%, 100%) |
|  |  |  | ≥20 to <50 | 15561 (42%, 44%) | 20160 (45%, 56%) | 35721 (43%, 100%) |
|  |  |  | ≥50 | 16711 (45%, 48%) | 18378 (41%, 52%) | 35089 (43%, 100%) |
|  |  |  | Total | 37282 (100%, 45%) | 45295 (100%, 55%) | 82577 (100%, 100%) |
|  | Cefazolin | sex | Female | 60304 (86%, 84%) | 11430 (83%, 16%) | 71734 (86%, 100%) |
|  |  |  | Male | 5088 (7%, 78%) | 1456 (11%, 22%) | 6544 (8%, 100%) |
|  |  |  | Total | 70156 (100%, 84%) | 13765 (100%, 16%) | 83921 (100%, 100%) |
|  |  | age group | <20 | 6907 (10%, 86%) | 1097 (8%, 14%) | 8004 (10%, 100%) |
|  |  |  | ≥20 to <50 | 30076 (43%, 85%) | 5425 (39%, 15%) | 35501 (42%, 100%) |
|  |  |  | ≥50 | 28421 (41%, 82%) | 6367 (46%, 18%) | 34788 (42%, 100%) |
|  |  |  | Total | 70156 (100%, 84%) | 13765 (100%, 16%) | 83921 (100%, 100%) |
|  | Ceftriaxone | sex | Female | 68095 (86%, 96%) | 2693 (69%, 4%) | 70788 (86%, 100%) |
|  |  |  | Male | 5835 (7%, 91%) | 574 (15%, 9%) | 6409 (8%, 100%) |
|  |  |  | Total | 78961 (100%, 95%) | 3878 (100%, 5%) | 82839 (100%, 100%) |
|  |  | age group | <20 | 7744 (10%, 98%) | 151 (4%, 2%) | 7895 (10%, 100%) |
|  |  |  | ≥20 to <50 | 34046 (43%, 97%) | 1017 (26%, 3%) | 35063 (42%, 100%) |
|  |  |  | ≥50 | 32154 (41%, 94%) | 2099 (54%, 6%) | 34253 (41%, 100%) |
|  |  |  | Total | 78961 (100%, 95%) | 3878 (100%, 5%) | 82839 (100%, 100%) |
|  | Ciprofloxacin | sex | Female | 28230 (78%, 91%) | 2739 (60%, 9%) | 30969 (76%, 100%) |
|  |  |  | Male | 3490 (10%, 83%) | 711 (16%, 17%) | 4201 (10%, 100%) |
|  |  |  | Total | 36212 (100%, 89%) | 4593 (100%, 11%) | 40805 (100%, 100%) |
|  |  | age group | <20 | 2785 (8%, 95%) | 135 (3%, 5%) | 2920 (7%, 100%) |
|  |  |  | ≥20 to <50 | 11923 (33%, 92%) | 991 (22%, 8%) | 12914 (32%, 100%) |
|  |  |  | ≥50 | 17023 (47%, 88%) | 2324 (51%, 12%) | 19347 (47%, 100%) |
|  |  |  | Total | 36212 (100%, 89%) | 4593 (100%, 11%) | 40805 (100%, 100%) |
| *S. aureus* SSTI | Clindamycin | sex | Female | 27693 (39%, 86%) | 4415 (36%, 14%) | 32108 (38%, 100%) |
|  |  |  | Male | 33954 (47%, 88%) | 4713 (38%, 12%) | 38667 (46%, 100%) |
|  |  |  | Total | 71696 (100%, 85%) | 12274 (100%, 15%) | 83970 (100%, 100%) |
|  |  | age group | <20 | 16882 (24%, 89%) | 2168 (18%, 11%) | 19050 (23%, 100%) |
|  |  |  | ≥20 to <50 | 24110 (34%, 88%) | 3288 (27%, 12%) | 27398 (33%, 100%) |
|  |  |  | ≥50 | 20717 (29%, 85%) | 3675 (30%, 15%) | 24392 (29%, 100%) |
|  |  |  | Total | 71696 (100%, 85%) | 12274 (100%, 15%) | 83970 (100%, 100%) |
|  | Erythromycin | sex | Female | 33051 (40%, 82%) | 7423 (41%, 18%) | 40474 (40%, 100%) |
|  |  |  | Male | 39070 (48%, 84%) | 7675 (42%, 16%) | 46745 (47%, 100%) |
|  |  |  | Total | 82082 (100%, 82%) | 18334 (100%, 18%) | 100416 (100%, 100%) |
|  |  | age group | <20 | 20891 (26%, 84%) | 4015 (22%, 16%) | 24906 (25%, 100%) |
|  |  |  | ≥20 to <50 | 28246 (34%, 83%) | 5688 (31%, 17%) | 33934 (34%, 100%) |
|  |  |  | ≥50 | 23045 (28%, 81%) | 5400 (30%, 19%) | 28445 (28%, 100%) |
|  |  |  | Total | 82082 (100%, 82%) | 18334 (100%, 18%) | 100416 (100%, 100%) |
|  | Methicillin | sex | Female | 21551 (38%, 65%) | 11716 (40%, 35%) | 33267 (39%, 100%) |
|  |  |  | Male | 27217 (48%, 68%) | 12721 (43%, 32%) | 39938 (46%, 100%) |
|  |  |  | Total | 56764 (100%, 66%) | 29642 (100%, 34%) | 86406 (100%, 100%) |
|  |  | age group | <20 | 13790 (24%, 67%) | 6804 (23%, 33%) | 20594 (24%, 100%) |
|  |  |  | ≥20 to <50 | 18069 (32%, 64%) | 10043 (34%, 36%) | 28112 (33%, 100%) |
|  |  |  | ≥50 | 16949 (30%, 69%) | 7616 (26%, 31%) | 24565 (28%, 100%) |
|  |  |  | Total | 56764 (100%, 66%) | 29642 (100%, 34%) | 86406 (100%, 100%) |
|  | Penicillin | sex | Female | 1991 (41%, 5%) | 38480 (47%, 95%) | 40471 (46%, 100%) |
|  |  |  | Male | 2897 (59%, 6%) | 43850 (53%, 94%) | 46747 (54%, 100%) |
|  |  |  | Total | 4889 (100%, 6%) | 82397 (100%, 94%) | 87286 (100%, 100%) |
|  |  | age group | <20 | 821 (17%, 3%) | 24086 (29%, 97%) | 24907 (29%, 100%) |
|  |  |  | ≥20 to <50 | 1678 (34%, 5%) | 32253 (39%, 95%) | 33931 (39%, 100%) |
|  |  |  | ≥50 | 2389 (49%, 8%) | 26057 (32%, 92%) | 28446 (33%, 100%) |
|  |  |  | Total | 4889 (100%, 6%) | 82397 (100%, 94%) | 87286 (100%, 100%) |
|  | Trimethoprim-sulfamethoxazole | sex | Female | 37062 (40%, 97%) | 1317 (33%, 3%) | 38379 (40%, 100%) |
|  |  |  | Male | 42942 (47%, 97%) | 1477 (37%, 3%) | 44419 (46%, 100%) |
|  |  |  | Total | 92029 (100%, 96%) | 3961 (100%, 4%) | 95990 (100%, 100%) |
|  |  | age group | <20 | 22426 (24%, 97%) | 745 (19%, 3%) | 23171 (24%, 100%) |
|  |  |  | ≥20 to <50 | 31486 (34%, 97%) | 936 (24%, 3%) | 32422 (34%, 100%) |
|  |  |  | ≥50 | 26151 (28%, 96%) | 1115 (28%, 4%) | 27266 (28%, 100%) |
|  |  |  | Total | 92029 (100%, 96%) | 3961 (100%, 4%) | 95990 (100%, 100%) |

* index isolate of each infection episode, excluding patients with only one isolate

Note: age/sex not available from Territory Pathology, so nitrofurantoin, trimethoprim and trimethoprim-sulfamethoxazole (for *E. coli*) not included in table. Totals include missing age/sex data but these missing categories are not shown.

Table S3. Number of events in each analysis.

|  | | Analysis 1 (number of index-susceptible infection episodes with subsequent resistant isolates [%]) | Analysis 2 (number of infection episodes that had subsequent recurrent infection [%]) | Analysis 2b (number of infection episodes that had subsequent recurrent infection [%]) | | Analysis 3 (number of infection episodes that had a subsequent *resistant** recurrent infection [%]) | Analysis 3b (number of infection episodes that had a subsequent *resistant* recurrent infection [%]) | |
| --- | --- | --- | --- | --- | --- | --- | --- | --- |
|  |  |  | Resistance combined^ | For each antibiotic separately | | Resistance combined^ | For each antibiotic separately | |
|  |  |  |  | Susceptible | Resistant |  | Susceptible | Resistant |
| *E. coli* UTI | Amoxicillin-clavulanate | 790/8191 [9%] | - | 17116/43650 [39%] | 4309/9936 [43%] | - | 1990/17102 [12%] | 2668/4306 [62%] |
|  | Ampicillin | 605/3897 [13%] | - | 8547/22872 [37%] | 12392/29952 [41%] | - | 1920/12366 [23%] | 10803/12366 [87%] |
|  | Cefazolin | 756/8319 [8%] | - | 17201/44133 [39%] | 4080/9025 [45%] | - | 1879/17177 [11%] | 2499/4079 [61%] |
|  | Ceftriaxone | 165/9997 [2%] | - | 19757/50254 [39%] | 1188/2223 [53%] | - | 442/19626 [2%] | 836/1184 [71%] |
|  | Ciprofloxacin | 111/5158 [2%] | - | 8303/20874 [40%] | 1428/2615 [55%] | - | 267/7831 [3%] | 1103/1320 [84%] |
|  | Nitrofurantoin | 21/1028 [2%] | - | 1329/3101 [43%] | 86/202 [43%] | - | 53/1329 [4%] | 39/86 [45%] |
|  | Trimethoprim | 63/521 [11%] | - | 756/1921 [39%] | 658/1383 [48%] | - | 111/756 [15%] | 548/658 [83%] |
|  | Trimethoprim-sulfamethoxazole | 61/553 [10%] | - | 802/2017 [40%] | 606/1274 [48%] | - | 123/801 [15%] | 486/606 [80%] |
|  | Resistant to 0 antibiotics | - | 15913/41177 [39%] | - | - | 2162/15913 [14%] | - | - |
|  | Resistant to 1 antibiotic | - | 2105/4745 [44%] | - | - | 1375/2105 [65%] | - | - |
|  | Resistant to 2 antibiotics | - | 3292/7429 [44%] | - | - | 2242/3292 [68%] | - | - |
|  | Resistant to 3 antibiotics | - | 117/255 [46%] | - | - | 103/117 [88%] | - | - |
|  | Resistant to 4 antibiotics | - | 25/47 [53%] | - | - | 22/25 [88%] | - | - |
| *S. aureus* SSTI | Clindamycin | 527/12590 [4%] | - | 14458/37470 [39%] | 3139/6899 [45%] | - | 1250/14253 [9%] | 2051/3086 [66%] |
|  | Erythromycin | 606/13279 [4%] | - | 16302/45442 [36%] | 4416/11109 [40%] | - | 1722/16281 [11%] | 2944/4412 [67%] |
|  | Methicillin | 743/8993 [8%] | - | 10890/32992 [33%] | 7063/17258 [41%] | - | 1767/10775 [16%] | 5699/6995 [81%] |
|  | Penicillin | - | - | 859/2603 [33%] | 17012/47000 [36%] | - | 466/858 [54%] | 16593/16991 [98%] |
|  | Trimethoprim-sulfamethoxazole | 279/15447 [2%] | - | 18828/50993 [37%] | 1008/2178 [46%] | - | 603/18530 [3%] | 569/993 [57%] |
|  | Resistant to 0 antibiotics | - | 2080/5623 [37%] | - | - | 848/2080 [41%] | - | - |
|  | Resistant to 1 antibiotic | - | 10720/31762 [34%] | - | - | 10243/10719 [96%] | - | - |
|  | Resistant to 2 antibiotics | - | 6506/16425 [40%] | - | - | 6355/6506 [98%] | - | - |
|  | Resistant to 3 antibiotics | - | 1242/2471 [50%] | - | - | 1201/1242 [97%] | - | - |
|  | Resistant to 4 antibiotics | - | 209/357 [59%] | - | - | 202/209 [97%] | - | - |

Analysis 1: predictors of resistance during an infection episode (Table 3)

Analysis 2: predictors of recurrent infection (Table 4); ^ including only amoxicillin-clavulanate, cefazolin, nitrofurantoin & trimethoprim for *E. coli* and clindamycin, methicillin, penicillin & trimethoprim-sulfamethoxazole for *S. aureus*

Analysis 2b: predictors of recurrent infection, separately for each antibiotic (Table S4)

Analysis 3: predictors of a resistant recurrent infection (Table 4); * resistance to ≥1 of the antibiotics; ^ including only amoxicillin-clavulanate, cefazolin, nitrofurantoin & trimethoprim for *E. coli* and clindamycin, methicillin, penicillin & trimethoprim-sulfamethoxazole for *S. aureus*

Analysis 3b: predictors of a resistant recurrent infection, separately for each antibiotic (Table S4)

Table S4. Predictors of recurrent infection and of a resistant recurrent infection, separately for each antibiotic (Analysis 2b and 3b respectively).

|  | | OR (95% CI); p-value; df* | |
| --- | --- | --- | --- |
|  |  | Recurrent vs no recurrent infection (Analysis 2b) | Resistant vs susceptible recurrent infection (Analysis 3b) |
| *E. coli* UTI | Nitrofurantoin-resistant | 1.01 (0.66-1.56); 0.95; 1,442 | 13.00 (6.37-26.37); <0.01; 714 |
|  | Number of infection episodes^A^ | 1.17 (1.12-1.24); <0.01 | 1.09 (1.02-1.16); 0.01 |
|  | History of recurrent infection^B^ | 1.28 (1.04-1.59); 0.02 | 0.96 (0.48-1.91); 0.90 |
|  |  | | |
|  | Amoxicillin-clavulanate-resistant | 1.08 (1.02-1.15); 0.01; 27,174 | 11.50 (10.42-12.7); <0.01; 12,537 |
|  | Female | 1 [reference] | 1 [reference] |
|  | Male | 1.44 (1.30-1.60); <0.01 | 1.42 (1.19-1.68); <0.01 |
|  | ≥20 to <50 years old | 1 [reference] | 1 [reference] |
|  | <20 years old | 0.95 (0.86-1.05); 0.33 | 0.96 (0.77-1.19); 0.70 |
|  | ≥50 years old | 1.15 (1.09-1.21); <0.01 | 1.14 (1.03-1.26); <0.01 |
|  | Number of infection episodes^A^ | 1.09 (1.09-1.10); <0.01 | 1.02 (1.00-1.03); 0.01 |
|  | History of recurrent infection^B^ | 1.55 (1.48-1.63); <0.01 | 0.91 (0.83-1.01); 0.07 |
|  |  | | |
|  | Ampicillin-resistant | 1.10 (1.04-1.15); <0.01; 27,198 | 25.99 (23.58-28.67); <0.01; 12,547 |
|  | Female | 1 [reference] | 1 [reference] |
|  | Male | 1.46 (1.32-1.61); <0.01 | 0.84 (0.70-1.01); 0.06 |
|  | ≥20 to <50 years old | 1 [reference] | 1 [reference] |
|  | <20 years old | 0.95 (0.86-1.05); 0.29 | 0.92 (0.74-1.13); 0.42 |
|  | ≥50 years old | 1.16 (1.10-1.22); <0.01 | 0.94 (0.85-1.04); 0.23 |
|  | Number of infection episodes^A^ | 1.09 (1.09-1.10); <0.01 | 1.02 (1.01-1.04); <0.01 |
|  | History of recurrent infection^B^ | 1.55 (1.48-1.63); <0.01 | 0.98 (0.89-1.08); 0.71 |
|  |  | | |
|  | Cefazolin-resistant | 1.10 (1.03-1.17); <0.01; 27,104 | 11.40 (10.33-12.60); <0.01; 12,508 |
|  | Female | 1 [reference] | 1 [reference] |
|  | Male | 1.45 (1.31-1.61); <0.01 | 1.38 (1.15-1.64); <0.01 |
|  | ≥20 to <50 years old | 1 [reference] | 1 [reference] |
|  | <20 years old | 0.96 (0.87-1.06); 0.40 | 0.97 (0.77-1.21); 0.78 |
|  | ≥50 years old | 1.15 (1.09-1.21); <0.01 | 1.15 (1.04-1.27); 0.01 |
|  | Number of infection episodes^A^ | 1.09 (1.09-1.10); <0.01 | 1.02 (1.01-1.04); <0.01 |
|  | History of recurrent infection^B^ | 1.55 (1.48-1.63); <0.01 | 0.89 (0.81-0.98); 0.02 |
|  |  | | |
|  | Trimethoprim-sulfamethoxazole-resistant | 1.18 (0.95-1.46); 0.13; 1,437 | 27.4 (18.33-41.80); <0.01; 710 |
|  | Number of infection episodes^A^ | 1.16 (1.11-1.23); <0.01 | 1.10 (1.02-1.19); 0.01 |
|  | History of recurrent infection^B^ | 1.28 (1.04-1.59); 0.02 | 0.79 (0.52-1.20); 0.27 |
|  |  |  |  |
|  | Trimethoprim-resistant | 1.21 (0.98-1.50); 0.08; 1,442 | 36.22 (23.77-56.54); <0.01; 713 |
|  | Number of infection episodes^A^ | 1.17 (1.11-1.23); <0.01 | 1.10 (1.03-1.18); 0.01 |
|  | History of recurrent infection^B^ | 1.27 (1.03-1.58); 0.03 | 0.73 (0.47-1.12); 0.16 |
|  |  | | |
|  | Ceftriaxone-resistant | 1.48 (1.32-1.67); <0.01; 26,672 | 83.15 (68.15-101.82); <0.01; 12,210 |
|  | Female | 1 [reference] | 1 [reference] |
|  | Male | 1.43 (1.29-1.59); <0.01 | 1.50 (1.09-2.05); 0.01 |
|  | ≥20 to <50 years old | 1 [reference] | 1 [reference] |
|  | <20 years old | 0.96 (0.87-1.06); 0.45 | 0.98 (0.58-1.59); 0.93 |
|  | ≥50 years old | 1.14 (1.08-1.20); <0.01 | 1.53 (1.24-1.88); <0.01 |
|  | Number of infection episodes^A^ | 1.09 (1.08-1.10); <0.01 | 1.02 (1.00-1.05); <0.01 |
|  | History of recurrent infection^B^ | 1.55 (1.48-1.63); <0.01 | 0.81 (0.66-0.98); 0.03 |
|  |  | | |
|  | Ciprofloxacin-resistant | 1.53 (1.35-1.73); <0.01; 10,444 | 147.23 (112.58-194.49); <0.01; 4,607 |
|  | Female | 1 [reference] | 1 [reference] |
|  | Male | 1.39 (1.21-1.59); <0.01 | 1.41 (0.95-2.07); 0.08 |
|  | ≥20 to <50 years old | 1 [reference] | 1 [reference] |
|  | <20 years old | 0.87 (0.72-1.04); 0.12 | 0.67 (0.28-1.47); 0.35 |
|  | ≥50 years old | 1.06 (0.98-1.16); 0.14 | 1.08 (0.81-1.45); 0.59 |
|  | Number of infection episodes^A^ | 1.10 (1.09-1.12); <0.01 | 1.03 (1.00-1.07); 0.08 |
|  | History of recurrent infection^B^ | 1.52 (1.40-1.65); <0.01 | 0.86 (0.65-1.13); 0.27 |
| *S. aureus* SSTI | Trimethoprim-sulfamethoxazole-resistant | 1.14 (0.98-1.31); 0.08; 19,882 | 38.04 (29.97-48.40); <0.01; 8,387 |
|  | Female | 1 [reference] | 1 [reference] |
|  | Male | 1.06 (1.00-1.13); <0.01 | 1.03 (0.83-1.29); 0.79 |
|  | ≥20 to <50 years old | 1 [reference] | 1 [reference] |
|  | <20 years old | 0.73 (0.67-0.79); <0.01 | 1.16 (0.83-1.62); 0.38 |
|  | ≥50 years old | 1.27 (1.19-1.36); <0.01 | 1.30 (1.02-1.67); 0.03 |
|  | Number of infection episodes^A^ | 1.11 (1.09-1.12); <0.01 | 1.03 (1.00-1.07); 0.07 |
|  | History of recurrent infection^B^ | 1.44 (1.35-1.52); <0.01 | 1.07 (0.85-1.33); 0.56 |
|  |  | | |
|  | Erythromycin-resistant | 1.19 (1.11-1.27); <0.01; 21,273 | 16.76 (14.88-18.90); <0.01; 8,964 |
|  | Female | 1 [reference] | 1 [reference] |
|  | Male | 1.06 (1.01-1.13); 0.03 | 1.08 (0.96-1.22); 0.19 |
|  | ≥20 to <50 years old | 1 [reference] | 1 [reference] |
|  | <20 years old | 0.73 (0.68-0.79); <0.01 | 0.85 (0.71-1.02); 0.08 |
|  | ≥50 years old | 1.29 (1.21-1.37); <0.01 | 1.23 (1.08-1.40); <0.01 |
|  | Number of infection episodes^A^ | 1.11 (1.09-1.12); <0.01 | 1.04 (1.02-1.07); <0.01 |
|  | History of recurrent infection^B^ | 1.44 (1.36-1.52); <0.01 | 1.11 (0.99-1.25); 0.08 |
|  |  |  |  |
|  | Methicillin-resistant | 1.29 (1.21-1.38); <0.01; 18,018 | 24.33 (21.51-27.56); <0.01; 7,391 |
|  | Female | 1 [reference] | 1 [reference] |
|  | Male | 1.11 (1.04-1.18); <0.01 | 0.89 (0.79-1.01); 0.06 |
|  | ≥20 to <50 years old | 1 [reference] | 1 [reference] |
|  | <20 years old | 0.74 (0.68-0.80); <0.01 | 0.85 (0.71-1.02); 0.09 |
|  | ≥50 years old | 1.32 (1.24-1.42); <0.01 | 1.00 (0.87-1.14); 0.95 |
|  | Number of infection episodes^A^ | 1.11 (1.09-1.12); <0.01 | 1.05 (1.02-1.07); <0.01 |
|  | History of recurrent infection^B^ | 1.46 (1.37-1.55); <0.01 | 0.90 (0.79-1.02); 0.10 |
|  |  | | |
|  | Clindamycin-resistant | 1.26 (1.16-1.37); <0.01; 17,232 | 21.01 (18.26-24.23); <0.01; 7,579 |
|  | Female | 1 [reference] | 1 [reference] |
|  | Male | 1.06 (1.00-1.13); 0.06 | 1.06 (0.92-1.22); 0.42 |
|  | ≥20 to <50 years old | 1 [reference] | 1 [reference] |
|  | <20 years old | 0.74 (0.68-0.81); <0.01 | 0.82 (0.65-1.03); 0.08 |
|  | ≥50 years old | 1.23 (1.15-1.32); <0.01 | 1.31 (1.13-1.53); <0.01 |
|  | Number of infection episodes^A^ | 1.09 (1.08-1.11); <0.01 | 1.04 (1.02-1.07); <0.01 |
|  | History of recurrent infection^B^ | 1.42 (1.34-1.52); <0.01 | 1.10 (0.96-1.27); 0.17 |
|  |  | | |
|  | Penicillin-resistant | 1.30 (1.14-1.49); <0.01; 21,273 | 34.33 (26.77-44.10); <0.01; 8,968 |
|  | Female | 1 [reference] | 1 [reference] |
|  | Male | 1.06 (1.01-1.12); 0.03 | 0.86 (0.68-1.09); 0.21 |
|  | ≥20 to <50 years old | 1 [reference] | 1 [reference] |
|  | <20 years old | 0.72 (0.67-0.78); <0.01 | 1.33 (0.88-2.03); 0.18 |
|  | ≥50 years old | 1.30 (1.22-1.39); <0.01 | 0.61 (0.47-0.78); <0.01 |
|  | Number of infection episodes^A^ | 1.11 (1.09-1.12); <0.01 | 1.02 (0.97-1.07); 0.54 |
|  | History of recurrent infection^B^ | 1.44 (1.36-1.53); <0.01 | 0.96 (0.76-1.21); 0.72 |

^A^ number of infection episodes per patient (including the current infection episode)

^B^ ≥1 infection episode in the previous 6 months

* model degrees of freedom (residual)

*4*

**Analysis 1**

recurrent infection of *4*

recurrent infection of *4*

<30d

<30d

>30d

<6m

<6m

<6m

>30d

*5*

*2*

*4*

*3*

*1*

*5*

*2*

*2*

*3*

recurrent infection of *1*

recurrent infection of *2*

recurrent infection of *3*

*1*

<30d

<30d

<30d

<30d

>30d

<30d

<30d

>30d

recurrent infection of *3*

recurrent infection of *2*

recurrent infection of *1*

>6m

>6m

*1*

*3*

**Analysis 2/2b**

**Analysis 3/3b**

Figure S1. A visualisation of one hypothetical individual’s data over time and how they contribute to each of the three analyses.

Isolates (solid/red circles=antibiotic-resistant, hollow/green circles=antibiotic-susceptible, black=not included in analysis) were grouped into infection episodes separated by >30 days (assume all isolates are the same organism / from the same anatomical site). Index isolates of each included infection episode labelled numerically.

Analysis 1, replicated for each antibiotic separately, included only infection episodes consisting of ≥2 isolates with a susceptible index isolate, censored at the first resistant isolate or last isolate if never resistant. Analysis 2 and 3 included all infection episodes, defined as resistant if ≥1 isolate was resistant (infection episode number in red), otherwise susceptible (green). Recurrent infection was defined as ≥1 infection episode in the 6 months after an infection episode and, in Analysis 3, was defined as resistant (red) if there was a resistant index isolate in ≥1 of these infection episodes, otherwise susceptible (green).


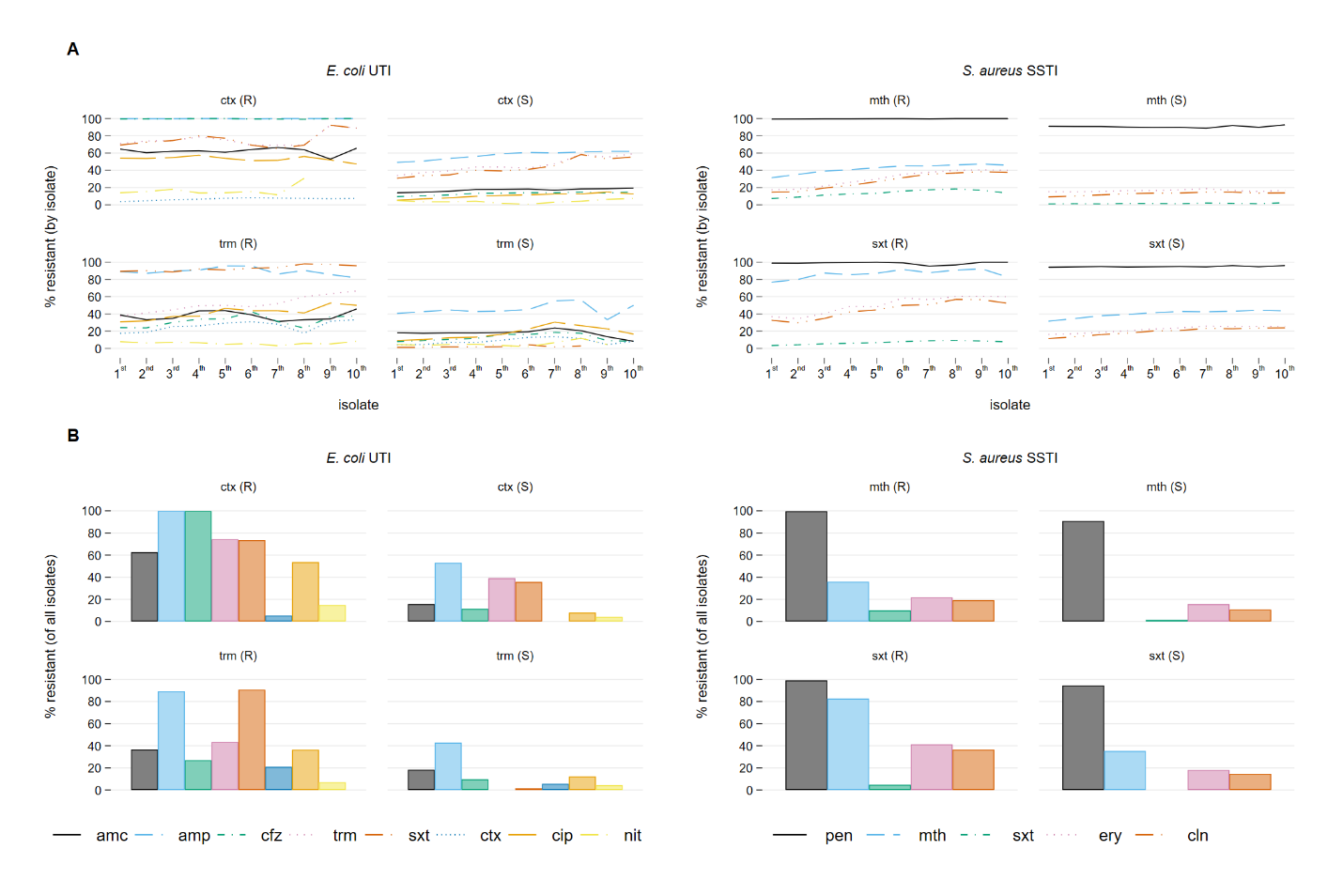


Figure S2. **A.** Percentage of isolates resistant to a second antibiotic dependent on susceptibility to the first, by isolate number per patient (up to the 10^th^ isolate, excluding patients with only one isolate). **B.** Percentage of isolates resistant to a second antibiotic dependent on susceptibility to the first, for all isolates combined (excluding patients with only one isolate).

Note: R: resistant; S: susceptible; amp: ampicillin; amc: amoxicillin-clavulanate; cfz: cefazolin; ctx: ceftriaxone; cip: ciprofloxacin; cln: clindamycin; ery: erythromycin; mth: methicillin (i.e., oxacillin or flucloxacillin); nit: nitrofurantoin; pen: penicillin; trm: trimethoprim; sxt: trimethoprim-sulfamethoxazole
